# Hepatitis C Virus Uses Host Lipids to Its Own Advantage

**DOI:** 10.3390/metabo11050273

**Published:** 2021-04-27

**Authors:** Malgorzata Sidorkiewicz

**Affiliations:** Department of Medical Biochemistry, Faculty of Health Sciences, Medical University of Lodz, 90-419 Lodz, Poland; malgorzata.sidorkiewicz@umed.lodz.pl

**Keywords:** HCV, lipoproteins, lipoprotein receptors, steatosis, hypolipidemia, lipid droplets, replication, very low density lipoprotein assembly

## Abstract

Lipids and lipoproteins constitute indispensable components for living not only for humans. In the case of hepatitis C virus (HCV), the option of using the products of our lipid metabolism is “to be, or not to be”. On the other hand, HCV infection, which is the main cause of chronic hepatitis, cirrhosis and hepatocellular carcinoma, exerts a profound influence on lipid and lipoprotein metabolism of the host. The consequences of this alternation are frequently observed as hypolipidemia and hepatic steatosis in chronic hepatitis C (CHC) patients. The clinical relevance of these changes reflects the fact that lipids and lipoprotein play a crucial role in all steps of the life cycle of HCV. The virus circulates in the bloodstream as a highly lipidated lipo-viral particle (LVP) that defines HCV hepatotropism. Thus, strict relationships between lipids/lipoproteins and HCV are indispensable for the mechanism of viral entry into hepatocytes, viral replication, viral particles assembly and secretion. The purpose of this review is to summarize the tricks thanks to which HCV utilizes host lipid metabolism to its own advantage.

## 1. Introduction

**Figure 1:**
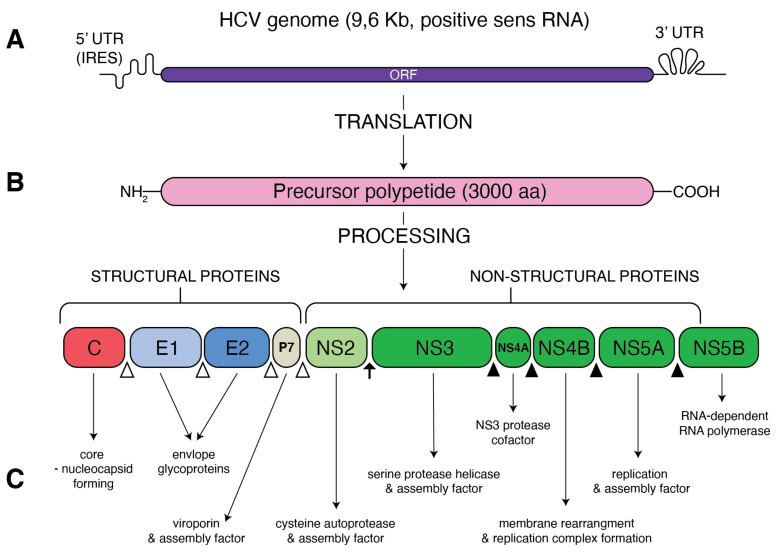
Schematic presentation of HCV genome and proteins. (**A**) A single open reading frame (ORF) of HCV genome is flanked by 5′ untranslated region (5′ UTR) that contains an internal ribosome entry site (IRES) and 3′ UTR. (**B**) IRES-mediated translation forms a precursor protein of 3000 aa, which is then processed into 10 different proteins. Structural proteins (core, E1, E2 and p7) are cleaved from precursor thanks to the cellular signal peptidase (empty triangles) The NS2–NS3 protease auto-cleaves itself (black arrow). The NS3 protease, with NS4A as a cofactor, cleaves the remaining non-structural proteins: NS3, NS4A, NS4B, NS5A and NS5B (black triangles). (**C**) Function of the resultant structural and non-structural proteins.

Hepatitis C virus (HCV), belonging to the family of Flaviviridae, genus *Hepacivirus*, is a small-enveloped virus with a single (+)-stranded RNA genome of approximately 9.5 kb (Figure 1). A large polyprotein encodes by the HCV genome is further processed into structural proteins: the core, envelope (E1 and E2), viroporin (p7) and non-structural (NS2, NS3, NS4A, NS4B, NS5A and NS5B) proteins. HCV shows enormous variability of its genomic sequence, and, thus far, eight genotypes and over 80 subtypes of HCV have been confirmed [1]. The story of HCV started to heat up over 30 years ago from the isolation of viral complementary DNA by Michael Houghton’s team [2]. Since 2005, the replicative HCV JFH1 strain, capable of forming infectious virions (HCVcc) from hepatoma cell lines in culture [3,4], has been used for further HCV studies. The continuous improvement of in vitro methods of HCV replication, such as the use of SEC14L2 that enables pan-genotype HCV replication in cell culture [5], still deepens our knowledge of this disease. Despite constant progress in our understanding of HCV life cycle and outstanding development of HCV diagnosis and treatment, HCV infection remains a serious health issue. It is estimated that over 71 million people worldwide are infected with HCV, accounting for 1% global population [6]. A majority of infected people do not realize that they have been infected, and the silently developing infection remains a major causative agent of chronic liver diseases. In turn, chronic hepatitis C (CHC) has been shown to be responsible for up to 25% of hepatocellular carcinoma cases worldwide [7]. Persistence and pathogenesis of such viral infection are caused by the ability of HCV to deregulate important host processes, mostly innate immunity and lipid metabolism. The association of HCV with lipid and lipoprotein metabolism has been observed clinically for many years. In particular, chronic hepatitis is considered to be the reason of liver steatosis and dyslipidemia [8,9].

HCV particles that are separated from the patients’ blood revealed a surprisingly low densities that was soon explained by their close association with lipoproteins [10]. It cannot be excluded that this association and related to this atypical density of HCV constituted an obstacle in identification of the factor responsible for the non-A, non-B hepatitis [2]. Over three decades of studies on hepatitis C virus have underlined the importance of host lipids and lipoproteins in the HCV life cycle. HCV particles together with lipoproteins forms complex particles called lipoviral particles (LVPs) [11]. Due to different lipoprotein components of LVP (e.g., cholesterol, TG, apoB, apoE, apoA-I and apoCs) circulating in the bloodstream, viral particles attach onto target cell membranes by sequential binding to lipoprotein receptors [12]. Then, HCV enters the cell by a clathrin-mediated endocytosis that involves other host molecules. Virus internalization is followed by translation and replication of HCV genome [13]. The interaction of newly synthetized HCV core protein with lipid droplets is essential for HCV genome replication and new viral particles’ assembly. It is presumed that HCV hijacks the very-low-density lipoprotein (VLDL) secretory pathway to become surrounded by lipoprotein and facilitate exit of progeny. The subsequent sections of this paper present in more detail the dependency and modification of lipid and lipoprotein metabolism which so vital for maintenance of the HCV infection.

## 2. Lipid Phenotype of Chronic HCV Patients

Even before isolation, the viral agent that caused non-A, non-B hepatitis was considered to be a factor that interferes with the host lipids, lipoprotein and cholesterol homeostasis. The lipid metabolism alteration favoring triglyceride accumulation in the liver was recognized as an outstanding feature in the liver biopsies of non-A, non-B hepatitis patients [8]. Immediately after the diagnostic tests for HCV became available, it was stated that hepatic steatosis is one of the characteristic histological features in the liver of HCV-infected patients. HCV-infected people have significantly higher prevalence of steatosis than in hepatitis B virus (HBV)-infected patients, and it is particularly strong in Genotype 3 infection, demonstrating a correlation between genetic variation of HCV and the ability to accumulate lipids [14,15]. The pathogenesis of HCV-induced steatosis is determined by viral factors as well as host factors. Hepatitis C infection is often connected with insulin resistance. It may be a result of impaired insulin signaling and activation of inflammatory markers (TNF-alpha and the suppressor of cytokine signaling family proteins), which in turn deregulates fatty acid synthesis in the host and causes hepatic steatosis. The pathogenesis of steatosis in patients infected with non-Genotype 3 HCV is frequently associated with an increased body mass index and visceral obesity, which suggests the genotype-specific origin of hepatic steatosis. That is why steatosis is sometimes separated right into “viral” and typical for Genotype 3 and “metabolic” in non-Genotype 3 infections [14,16]. Experimental results show that HCV recruits and utilizes host lipid droplets to perform own replication. HCV core protein interaction with host lipid droplets seems to be an important factor in this process [17,18,19]. Thanks to the host diacylglycerol acyltransferase1 activity, the core protein is localized on the surface of lipid droplets (LD). LD–core complex deregulates lipids turnover, taking part in the development of steatosis. Additionally, HCV infection induces activation of sterol regulatory element binding protein (SREBP), the transcription factor responsible for lipogenesis [20,21,22]. Downregulation of carnitine palmitoyltransferase expression may additionally increase steatosis by inhibition of beta-oxidation [23]. Overproduction of lipid droplets has also been observed in HCV-transfected cells. In a transgenic murine model of viral-related steatosis, the HCV core protein modified VLDL secretion by exerting an influence on microsomal triglyceride transfer protein (MTP). Inhibition of MTP, essential for normal assembly and secretion of VLDL, results in liver steatosis and hypolipidemia [24,25]. The next important cause of steatosis development may be connected with the expression of microRNAs that play an essential role in controlling the metabolism of cholesterol and fatty acid. It was shown that serum level of miR-122 and miR-34a is correlated with the grade of steatosis in chronic HCV [26]. Clinical evidence indicates that HCV infection is not only connected with lipid metabolic pathways in hepatocytes but also alters the metabolism of circulating lipoprotein. Paradoxically, hypocholesterolemia was frequently described in HCV-infected patients despite the important role of lipoproteins in the propagation of HCV infection. At first glance, it is not certain whether this phenomenon is the result of hepatic damage caused by inflammation or is a specific effect of HCV infection [27]. The latter conception is supported by studies that showed significantly lower cholesterol level in HCV-infected patients as compared to HBV infected ones. It has been documented by many investigators that HCV infection affects total serum cholesterol, LDL cholesterol and ApoB [9,28,29]. It has been suggested that HCV reduces the activity of microsomal triglyceride transfer protein (MTP), which causes decreased loading of lipids onto apoB [30]. Enlarged pre-secretory apoB degradation leads to development of hypobetalipoproteinemia. This, in turn, is related to impaired VLDL secretion and, consequently, decreased LDL level in sera. Inhibition of VLDL secretion not only decreases serum cholesterol and triglycerides but also increases the triglycerides storage in the liver [31]. That is why serum cholesterol level is frequently inversely related to the degree of steatosis. Interestingly, HCV-related hypocholesterolemia resolves with successful anti-viral treatment and that is not observed in non-responders. It has also been shown that higher cholesterol and LDL levels before the treatment are associated with strong possibility of obtaining a sustained virologic response (SVR), and a lower pretreatment cholesterol level is a predictor of treatment failure [32,33]. Hypolipidemia, with a decreased LDL cholesterol level, also appears in acute hepatitis C infection, and the resolution of infection results in an increase of this parameter [34]. The effect of HCV infection on patients’ serum lipid profile strongly suggests the connection between host lipoprotein metabolism and the viral life cycle. What seems to be of special interest is the ability of HCV to mimic lipoprotein particles and all consequences connected with this phenomenon.

## 3. Resemblance between HCV Particles and Lipoproteins

**Figure 2:**
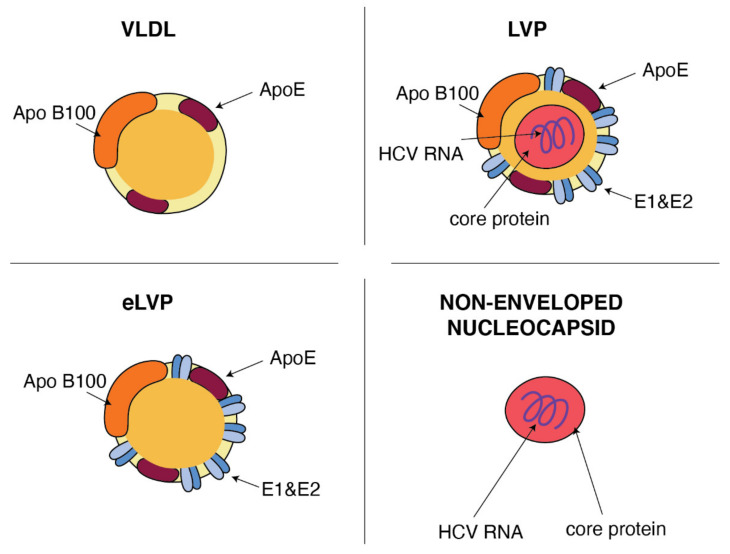
Similarities between VLDL and HCV particles. ApoB and ApoE apolipoproteins classically associated with host VLDL particles are very useful components of HCV particles: lipo-viral particles (LVP) and empty lipo-viral particles (eLVP). An additional component that appears in the lipid envelope of these two HCV particles is a heterodimer composed of viral E1 and E2 envelope glycoproteins. Capsid, formed by the viral core protein, containing the positive HCV RNA strand may be surrounded by the lipid envelop with embedded E1 and E2 glycoproteins, such as in the case of LVP, or may form a non-enveloped viral particle also presented in the blood of HCV-infected patients.

In general terms, the HCV particle should consist of a nucleocapsid containing the single-stranded RNA genome connected to the viral core protein and an envelope membrane containing the surface viral glycoproteins E1 and E2 [35]. However, viral particles purified from sera of infected patients revealed that the structure of HCV is much more complex and HCV particles are extremely heterogeneous [36]. A sub-population of HCV particles in sera is known as a mix of infectious and noninfectious particles with spherical morphology of various sizes with a diameter in the range 40–70 nm and density from 1.20 to 1.03 g/cm^3^. This heterogeneity and the high buoyant density results from the association of HCV with apolipoprotein B (ApoB), including lipoproteins: very-low-density lipoproteins (VLDL) and low-density lipoproteins (LDL) [11,37,38]. The classification of lipoproteins is based on their density, i.e., different contents of cholesteryl esters (CE), triglycerides (TG), free cholesterol, phospholipids and apolipoproteins, which help with assembly, transport and metabolism of these particles [39]. Lipoproteins play an important role in lipid transportation through the bloodstream and the delivery of lipids to the target cells. VLDL, the most abundant lipoprotein, produced in the liver [40], consists of the central core that is rich of triglycerides and cholesterol esters and is surrounded by phospholipids and cholesterol envelope with constant presence of ApoB and apolipoprotein E (ApoE). The associations of HCV particles with lipoproteins were verified by immunoprecipitation of the HCV RNA including fractions with anti-apolipoprotein antibodies against ApoB or ApoE. ApoB was spotted at a higher density of 1.06–1.07 g/mL in people suffering from chronic HCV in comparison to healthy volunteers in whom apoB was detected at a density <1.06 g/mL [41]. In the endogenous transport pathway, VLDL particles [42] carry both triglycerides and cholesterol from the liver to the peripheral tissues. Hydrolysis of the TG core delivers free fatty acids mainly to muscle and adipose tissues. Most of the resulting TG-depleted VLDL remnant (called IDL) are cleared directly from the plasma by the liver. The remaining part is converted by the lipolysis [43] to CE-rich LDL particles. They deliver cholesterol to peripheral tissues and then they are received by the LDL receptors (LDLR) and internalized via a clathrin-dependent pathway. Interestingly, using of LDLR and clathrin also represents important parts of a multi-step process of HCV entry into hepatocytes (see Section 4). HCV particles in the serum are presented as a mix of complete low-density infectious, so-called lipo-viral particles (LVP), nonenveloped nucleocapsids and a great amount of empty lipoviral particles (eLVP) that are also described as nucleocapsid-free sub-viral particles (Figure 2). LVP is known as a hybrid particle that consists of viral components and cell-derived triglyceride-rich lipoproteins, that include some apolipoproteins (ApoE, ApoB, ApoCI, ApoCII and ApoCIII). In comparison to lipoproteins of the same density, LVP have 30 times more TG per particle. This is probably because of a heavier non-lipid load consisting of HCV RNA and the core protein [44]. An analysis of sucrose gradients in the serum of infected people showed that HCV RNA is distributed over a variety of densities from 1.20 to 1.03 g/cm^3^ [34]. Virions with a very low buoyant density (range 1.10–1.14 g/mL) have shown the highest infectivity [45]. The contribution of LVP to total HCV viral load is highly dynamic and affected by lipoprotein metabolism [44]. The presence of nonenveloped HCV nucleocapsids in the serum of HCV-infected patients probably contributes to persistent infection and HCV escape from immune surveillance [46]. Apart from infectious LVP, envelope E1 and E2 proteins were found on the surface of lipoprotein particles devoid of infectious nucleocapsids, also known as empty LVP. Their presence may contribute to the pathology of hepatitis C [44]. The presence of apolipoproteins on the surface of LVP and empty LVP can mask viral epitops and allows HCV to avoid anti-HCV neutralizing antibodies [47].

The ultrastructural study by Piver et al. [48] of serum-derived HCV provides additional evidence confirming the existence of both the lipo-viral particles and lipoprotein-like particles likely to be an equivalent of nucleocapsid-free sub-viral particles. Not only HCV particles isolated from the plasma of chronically infected patients [44] but also particles from primary culture of adult human hepatocytes (HCVpc) [49] show similarities with VLDL concerning the structure. Additionally, the analysis of HCVcc confirmed some observations made in relation to lipo-viral particles in the serum and concerning density and size heterogeneity of these particles [50]. Moreover, the inhibition of MTP prevents HCVcc producing, as well as silencing of apolipoproteins apoE and apoB [51,52]. However, some dissimilarities are found in the composition of LVP in the serum of HCV-infected people and particles formed in culture. Immunoprecipitation by anti-apoB antibodies is more efficient in the case of serum–derided particles than in the case of HCVcc. The average density of HCVcc is higher as compared to LVP from human serum (1.10 vs. 1.05 g/cm^3^) [3,53]. It is probable that we can observe here the differentiation in the lipid content of serum LVP and the particles created in cell culture. Huh7 cells secrete more LDL-sized than VLDL-sized particles [54]. It is easy to deduce that having the structure similar to that of lipoprotein, HCV become more effective at transporting infection throughout the body and at entering the target cells.

## 4. Lipid-Dependent HCV Entry

**Figure 3:**
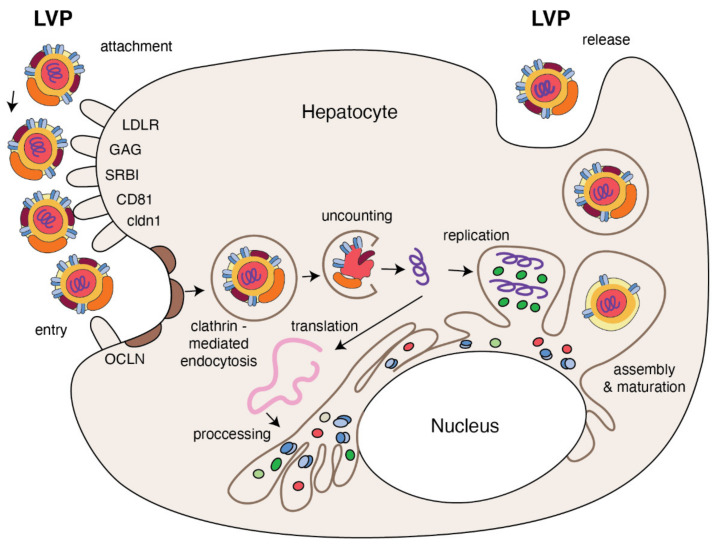
The replication cycle of HCV. Lipo-viral particle binds to the host cellular receptors such as LDLR, SRB1, CD81, claudin1 and others (please refer to the main text). Following attachment, the virus enters the target cell via clathrin-mediated endocytosis. Upon entry, HCV RNA is released into the cytoplasm and is directly translated via an IRES at the ER. A polyprotein precursor is then processed by both viral and host proteases. Non-structural viral proteins and host factors form a membranous web and replication complexes that are used for synthesis of multiple HCV RNA copies. Viral progenies are packed, forming nucleocapsid that is enriched by viral envelope glycoprotein. The access to the lipoprotein pathway provides conditions for incorporation of neutral lipids and apolipoproteins and formation of the mature lipo-viral particle released from hepatocytes by exocytosis.

HCV entry into human hepatocytes is a multi-step process (Figure 3) in which many host factors are involved including glycoaminoglycans (GAGs), low density lipoprotein receptors (LDLr), scavenger receptors class B type I (SR-BI), tetraspanin CD81, the tight junction proteins, claudin-1 (CLDN1), occludin (OCLN), receptor tyrosine kinases (RTKs), the Niemann–Pick C1-like 1 (NPC1L1) and epidermal growth factor receptor (EGFR) [55,56]. The first step, HCV entry from the bloodstream into the target cell, depends on the viral attachment with GAGs and LDLr present on the basolateral membrane of the hepatocyte. It has been demonstrated that HCV binds to the GAGs present on heparan sulfate (HS) proteoglycans (HSPGs), and syndecan 1 and 4 are involved in this process [57,58]. ApoE mediates HCV attachment through specific interactions with HSPG [59,60] and enhances HCV entry by interaction with LDLr [61]. HCV envelope E1 glycoprotein interacts with apoE via its N-terminal ectodomain to facilitate HCV entry via LDLr [62]. The physiological role of LDLr is to regulate transport of cholesterol-rich LDL intracellularly via clathrin-mediated endocytosis [63]. In the case HCV infection, LDLr function seems to be more complex [64]. In primary human hepatocytes, a peptide inhibitor of LDL binding to LDLr inhibits HCV infection, as does treatment of hepatocytes with monoclonal antibodies against LDLr or LDL [65]. It has been suggested that, if HCV and LDL are competitive for the cellular LDLr, LDL concentration in HCV-infected patients may regulate the binding of HCV to target cells [66]. This could explain why high LDL cholesterol is a predictor of treatment response [67]. Following an initial interaction with GAGs and LDLr, in the next step of entering, HCV uses SR-BI, the major receptor of high-density lipoprotein (HDL) that binds also VLDL and LDL particles [68,69]. The interaction between SR-B1 and HCV is probably responsible for the dissociation of lipoproteins from the surface of HCV particles due to SR-B1-mediated cholesterol transfer. It results in an alteration of E2 glycoprotein conformation that helps in HCV interaction with CD81 [70]. However, other results suggest that HCV binding with SR-B1 is mediated by ApoE [71,72].

The interaction between HCV and the tetraspanin CD81, in turn, is necessary to begin the HCV internalization. HCV-CD81 complex moves toward tight junctions and interacts with tight junction proteins CLDN1 and OCLN that cause viral internalization in cholesterol-rich microdomains via clathrin-mediated endocytosis. Additional host factors, such as RTKs and NPC1L1, are known as regulatory cofactors in this process. The latter helps with viral entry by cholesterol regulation, while RTKs by signal transduction induce CD81–CLDN1 association and membrane fusion [56,73,74]. The study performed on the three-dimensional polarized hepatoma system confirmed that initially HCV particles colocalized with early entry factors at basolateral membranes are then accumulated at the tight junction due to the direct interaction between E1/E2 and OCLN, in an actine-dependent manner [75]. The same study showed that EGFR plays an important role in recruitment of clathrin-coated resides necessary for HCV internalization. HCV internalization in clathrin vessel induces fusion between viral glycoproteins and early endosomes and acidification of vacuole [76]. This process is additionally stimulated by ApoC-I apolipoprotein that, through interaction with viral glycoproteins, also increases HCV infectivity [77]. After this pH-dependent fusion between viral and target membranes, HCV capsid is delivered into the cytosol, destroyed and the resultant HCV RNA is ready for the main part of replication. 

## 5. Host Factors Contribution to the Efficient HCV Replication

Replication is the next complex process of the viral life cycle that is facilitated by interaction of HCV with various lipid-related factors. After HCV entry, a disrupted viral capsid releases single-stranded RNA genome of positive polarity to the cytoplasm. Directly afterwards, HCV RNA is translated on the rough endoplasmic reticulum (ER). 5′- and 3′-untranslated regions that flanked the HCV genome are necessary for both viral transcription and replication. HCV translation is strictly regulated by internal ribosome entry site (IRES), which is located in the 5′ UTR [78]. HCV RNA translation is initiated by binding of the 5′ UTR IRES to the ribosomal 40S subunit [3]. In addition, in the 5′ UTR, two sites for microRNA-122 binding were found. The highly abundant liver-specific microRNA-122 (miRNA-122) controls translation and transcription of many genes connected with lipid metabolism [79]. Generally, microRNAs negatively influence on cellular mRNA, but, here, the binding of miR-122 to the 5′ noncoding region of the HCV genome [80] results in upregulation of viral RNA levels [81]. This result is connected with the promotion of HCV translation through the maintaining of proper secondary structure of IRES as well as through the protection of 5′ terminus of HCV RNA from degradation by host exonucleases [82]. The primary translation product is a ~3000 amino acid long polyprotein precursor that is then cleaved into mature viral proteins by host and viral proteases. This process is followed by colocalization of non-structural proteins through the anchoring of these proteins on membranes that are used as a place of viral replication. Geranylgeranyl pyrophosphate, the product of cholesterol biosynthetic pathway, is known as the factor responsible for the protein prenylation, the process that is required for protein membrane-anchoring. It was shown that host proteins geranylgeranylation may influence the HCV infection [83]. HCV RNA replication requires geranylgeranylated host proteins, as was found for prenylated host FBL2 protein that binds to NS5A [84]. It may explain how NS5A protein colocalizes in membrane, where HCV RNA replication occurs. Newly synthetized HCV proteins, together with host factors, induce the formation of special platform, the so-called membranous web (MW), composed of vesicles as well as cytosolic lipid droplets (LD) [85]. HCV, similar to other positive-sense RNA viruses, remodels intracellular membranes in host cells to form an effective replication site. For this reason, HCV nonstructural proteins function to modify the endoplasmic reticulum and cytosolic lipid droplets to form double membrane vesicles (DMVs) containing HCV replication complexes [85] where viral RNA is protected from host RNAses and sensors of the innate immunity that would respond to double-stranded RNA [85,86]. HCV replication complexes contain HCV RNA, the non-structural viral proteins necessary for replication and the newly synthesized viral RNA [87,88]. Massive rearrangement of host lipid structure is indispensable to form efficient HCV replication complexes. The study on lipin 1 [89], an enzyme that forms diacylglycerol in the cytoplasm and regulates the expression of genes involved in lipid metabolism, showed the involvement of this enzyme in generation of membranous web. It suggests the capacity of lipin1 to modify lipids and contribute to the formation of MW. Resulting membranous is composed mostly from double-membrane vesicles. These cholesterol-enriched membranes are formed by lipid transfer proteins such as Nieman–Pick type C1 protein that requited cholesterol to the membranous replication organelle. It was observed that NPC1 inhibition alters cholesterol transport and reduces HCV replication [90]. Apart from the structural changes, HCV infection influences the lipid composition of membranes affecting the subcellular distribution of lipid kinase [91,92]. This redistribution is necessary for increasing cholesterol and sphingolipids contents of membranes. It was demonstrated that HCV replication occurs in cholesterol- and sphingolipids-rich membranes [93]. The biogenesis of the membranous web that is induced by the viral NS4B and NS5A requires the activity of cellular lipid kinases belonging to the phosphatidylinositol-4-kinase (PI4K) III family [94]. HCV NS5A protein activates PI4K III alpha enzyme that gives the production of phosphatidylinositol-4-phosphate (PI4P) in replication complexes [95]. HCV modulates phosphatidylinositol signaling pathway in the way that cause the exchange of phosphatidiloinositol-4-phosphate with cholesterol in replication organelles. It was shown that also oxysterol-binding protein with phosphatidylinositol-4-kinase contribute to the cholesterol enrichments of MW [96]. The next study revealed that phosphatidylinositol transfer protein Nir2 can replace phosphatidylinositol through the interaction with VAPS and forms condition for continuous HCV replication [97]. It was also observed that, to maintain an efficient HCV replication level in cell culture, adoptive mutations are necessary to regulate the activity of phosphatidylinositol-4-kinase [98]. HCV infection is able to alter lipidomic profile of target cells. A lipidomic study showed that significant changes in the lipid composition are required for efficient HCV replication [99]. HCV infection decreases a ratio of neutral to membrane lipids in target cells; concerning glycerolophospholipids, increased contents of longer fatty acids was observed. On the other hand, fatty acid content of the host cell may in turn influence HCV replication. Supplementation of polyunsaturated fatty acids inhibits the process [100] by alteration of the membranous web, whereas monounsaturated fatty acids stimulate HCV replication [83]. Going back to the process of HCV RNA multiplication, the positive RNA genome is the template for the negative HCV RNA strands in the reaction catalyzed by viral protein NS5B, the RNA-dependent RNA polymerase. The newly synthesized strands become the templates to positive HCV RNA strands synthesis. Translation from newly synthetized positive RNA strands yields the production of new viral proteins. Following the accumulation of positive strands of HCV RNA and viral structural proteins, the assembly of new HCV particles is ready to start.

## 6. HCV Hijacks the VLDL Secretory Pathway for Progeny Formation

The special membranous matrix formation is not only necessary for HCV RNA replication, rearrangement of lipid membranes and lipid droplets in host cell is also a prerequisite for a successful viral assembly. Probably, to avoid competition for using HCV RNA, HCV replication is separate in time and space from the formation of nucleocapsid [101]. After translation and processing, the structural HCV proteins core, E1 and E2 are localized in distinct places within the cell. The newly synthetized HCV core protein, responsible for forming the viral capsid, is first localized on the surface of cytosolic lipid droplets (LDs) mainly through two amphipathic helices located within the central domain of core protein [102]. This process depends on host diacylglycerol acyltransferase 1 (DGAT1), an enzyme that synthesizes triglycerides in the endoplasmic reticulum (ER) and is involved in LD and VLDL morphogenesis [19,103]. This localization of core protein enables the recruitment of other cellular and viral components necessary for viral assembly [104] especially newly synthetized HCV RNA from MW and envelope E1 and E2 proteins from ER. The positively charged N-terminal residue of core is implicated in HCV RNA binding and forming of the nucleocapsid [105]. 3’ UTR was identified as a cis-acting element responsible for the HCV RNA encapsidation [106]. The viral envelope is acquired by budding into the ER at sites of lipoprotein where lipidation might occur via interaction between the virion and lipoproteins. The NS5A protein is an important factor of assembly of viral particles on the lipid-core stage [107]. N-terminal region of NS5A forms a dimer that binds RNA [13] and the phosphorylation of NS5A helps with efficient interaction with core protein and virion assembly [107]. Due to the newly-established live cell imaging system it was possible to visualize some aspects of HCV assembly [101]. According to this observation, lipid droplets are wrapped by double ER membranes and then linked with the sites of HCV replication-double membrane vesicles. E2-NS5A structures were visualized in close proximity to LD that seems to correspond to HCV assembly sites. This study confirms that viral replication and viral assembly are closely related not only in time but also in space [101]. In the next step, glycoprotein envelope proteins E1 and E2 originated from the endoplasmic reticulum can join with these lipid-capsid complexes [108]. It was suggested that E1 and E2 secretion depends on the assembly with ApoB-containing lipoproteins [109]. Further studies showed that ApoE is require for proper assembly of HCV particles [110,111,112]. In addition, ApoE was found necessary for viral l, cell-to-cell transmission [113]. It means that ApoE not only participate in HCV attachment and entry but also increase HCV infectivity [72]. Interestingly, Fukuhara et al. [114,115] showed that that apoB and ApoE may redundantly participate in HCV assembly like alternative factors that contain amphipathic alpha-helice. The final maturation of HCV particles is strictly associated with host VLDL biogenesis pathway. According to the study of Neumann et al. [116], the liver of infected patients can produce 10^12^ virions per day. Simultaneously, VLDL secretory pathway is able to produce 10^18^ particles. Formation of VLDL begins with the process of ApoB-100 lipidation that is catalyzed by microsomal triglyceride transfer protein and then followed by the fusion of pre-VLDL particles with Apo-E [42]. The primary action of VLDL assembly requires the co-translational lipidation of Apo-B by microsomal triglyceride transfer protein (MTP) generating a pre-VLDL particle. The pre-VLDL then converts into VLDL by fusing with triglyceride rich droplets, probably in post-ER compartments [117]. MTP seems also to be responsible for the incorporation of Apo-E and Apo-CIII on the surface of LDs [118]. Interestingly, all the proteins required for VLDL assembly were found in HCV replication complexes isolated from human hepatoma cells [119]. The newly assembled immature HCV virions probably fuse with the pre-VLDL particle prior to or during the second maturation step of VLDL generating. Then, HCV particles are released into the extra-cellular milieu via the constitutive VLDL secretory route in order to produce a mature LVP. Therefore, it looks that HCV hijacks the VLDL-producing machinery for the purpose of progeny formation. However, the exact mechanism of HCV association with host lipoproteins has not been fully elucidated to date and remains controversial [118]. One model, presented above, suggests direct incorporation of nascent virion into lipoproteins intracellularly, with the involvement of VLDL biogenesis pathway. On the other hand, some studies suggest that lipidation of HCV particles is an extracellular process. The presence of HCV particles associated with ApoB48, a lipoprotein specific for chylomicrons, suggests that maturation of HCV particles may occur outside hepatocytes [120]. Moreover, it was recently demonstrated that the density of HCV particles alters after incubation in lipid-rich medium [121]. It means that the mode of HCV lipidation and association with lipoproteins is still unclear. What we know beyond doubt is the fact that HCV particles are unique by their structure and close association with the host lipids and lipoproteins.

## 7. Conclusions

Our understanding of HCV life cycle has developed over the past three decades of studies. The most intriguing results accelerating with the development of viral culture systems highlight the role of host lipids and lipoproteins at all steps of HCV life cycle. Simultaneously with these findings, clinicians have revealed that infection with HCV is connected to serious lipid disorders such as liver steatosis and hypocholesterolemia. More and more evidence suggests that HCV hijacks host lipid metabolism to maintain an effective viral infection and escape the recognition by the host immune system. This review is an attempt to explain how utilization of host lipid and lipoprotein components may mediate attachment and entrance of HCV to target cells and help with viral replication and production of progeny. A better understanding of the intimate relationship between the HCV live cycle and host lipid and lipoprotein metabolism could bring new insight into the HCV survival mechanism, promote development of effective treatment, ensure global control of the HCV infection and help to avoid its fatal consequences.
